# A redetermination of the structure and Hirshfeld surface analysis of poly[di­aquadi-μ-hydroxido-tetra­kis­(μ-nicotinato *N*-oxide)tricopper(II)]

**DOI:** 10.1107/S2056989021002000

**Published:** 2021-02-26

**Authors:** Masoud Mirzaei, Maryam Bazargan, Pouria Ebtehaj, Joel T. Mague

**Affiliations:** a Department of Chemistry, Faculty of Science, Ferdowsi University of Mashhad, 9177948974, Mashhad, Iran; b Department of Chemistry, Tulane University, New Orleans, LA 70118, USA

**Keywords:** crystal structure, nicotinic acid N-oxide, copper, hydrogen bond

## Abstract

The product obtained from the reaction of pyridine-2,3-di­carb­oxy­lic acid and hydrated copper(II) chloride in hot aqueous NaOH solution was determined by low temperature X-ray diffraction to be [Cu_3_(C_6_H_4_NO_3_)_4_(OH)_2_(H_2_O)_2_]_*n*_ or [Cu_3_(μ-OH)_2_(μ-nicNO)_4_(H_2_O)_2_]_*n*_ (nicNO is pyridine-3-carboxyl­ate *N*-oxide), a structure obtained from room temperature data and reported previously. The present determination is improved in quality and treatment of the H atoms. A Hirshfeld surface analysis of the inter­molecular inter­actions is presented.

## Abstract 2

The structure of the product obtained from the reaction of pyridine-2,3-di­carb­oxy­lic acid and hydrated copper(II) chloride in hot aqueous NaOH solution was shown to be {[Cu_3_(μ-OH)_2_(H_2_O)_2_(μ-nicNO)_4_]}_*n*_ (nicNO is pyridine-3-carboxyl­ate *N*-oxide), which evidently arose from deca­rboxylation and oxidation of the reactant acid. The structure has been reported previously using room-temperature data. Here, a more complete description of the inter­molecular inter­actions, including a Hirshfeld surface analysis, is reported.

## Chemical context   

*N*-oxidation of the pyridine ring can significantly increase its electron-donating ability because the charge-polarized pyridine-*N*-oxide moiety can donate three pairs of electrons while a neutral nitro­gen atom in pyridine only gives one pair of electrons. Therefore, it is expected that *N*-oxidation can increase the coordination capacities and flexibility of the ligand. Metal complexes of pyridine-*N*-oxide ligands have been found to be particularly useful in the selective adsorption and separation of gases (CO_2_ over CH_4_) and as anti-HIV and luminescent agents (Noro *et al.*, 2015 ▸; Xiong *et al.*, 2014 ▸; Balzarini *et al.*, 2005 ▸; Lis *et al.*, 2002 ▸). These features have motivated our inter­est in the chemistry of carb­oxy­lic acid derivatives of pyridine-*N*-oxide for investigating the influence of the *N*-oxide moiety on the coordination mode(s) in the crystal lattice (Mirzaei *et al.*, 2020 ▸; Hosseini-Hashemi *et al.*, 2018 ▸, 2019 ▸; Baza­rgan *et al.*, 2016 ▸, 2020 ▸; Mirzaei, Eshtiagh-Hosseini, Baza­rgan *et al.*, 2015 ▸; Shahbazi *et al.*, 2017 ▸; Mirzaei, Eshtiagh-Hosseini, & Baza­rgan, 2015 ▸). Here, we report the isolation and X-ray crystal structure of the coordination polymer [Cu_3_(μ-OH)_2_(H_2_O)_2_(μ-nicNO)_4_]_*n*_ (**1**) (nicNO is pyridine-3-carboxyl­ate *N*-oxide) as the unexpected product from the reaction of pyridine-2,3-di­carb­oxy­lic acid with hydrated Cu^II^ chloride. It appears that oxidation and deca­rboxylation of the starting acid occurred during the reaction, as has been seen previously (Hosseini-Hashemi *et al.*, 2018 ▸; Mirzaei, Eshtiagh-Hosseini *et al.*, 2015 ▸). During the course of this work, we found two prior reports of this structure [NICTCU (Knuutilla, 1981 ▸) and NICTCU01 (Kang *et al.*, 2020 ▸)], both obtained with room-temperature data. Overall, the present structure is the same as the previous ones, but with some differences in metrical parameters as a result of the lower temperature of the data collection used here, a lower *R* value [0.0250 for all reflections (3592) *vs* 0.0416 for 2525 with *I* > 3*σ*(*I*) in NICTCU and 0.0538 for 3349 with *I* > 2*σ*(*I*) in NICTCU01. The present structure has slightly better s.u.’s on all derived parameters than obtained for NICTCU and significantly better ones than those obtained by Kang *et al.*. One deficiency of the NICTCU structure is the free refinement of hydrogen-atom parameters, a risky procedure with room-temperature data when heavy atoms are present, which led to C—H distances for the aromatic rings varying from 0.97 (2) to 0.84 (3) Å and O—H distances of 0.77 (3) to 0.41 (4) Å, the last three being particularly unrealistic. In addition, there was no absorption correction despite a linear absorption coefficient of 2.422 mm^−1^. Kang *et al.* performed an absorption correction and treated hydrogen atoms appropriately, but with an *R*_int_ of 0.0780 their data are clearly of poorer quality than in the present case (*R*_int_ = 0.0208).
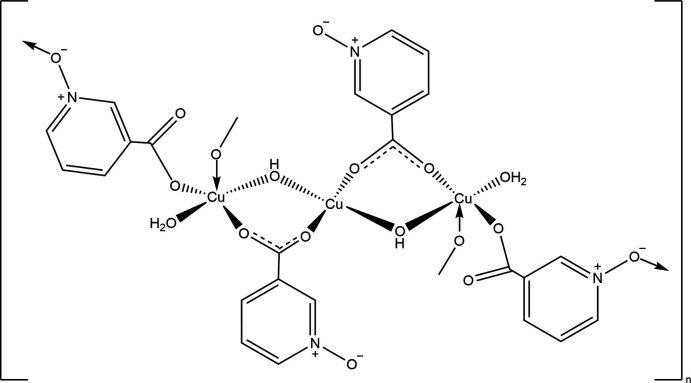


## Structural commentary   

**Figure 1:**
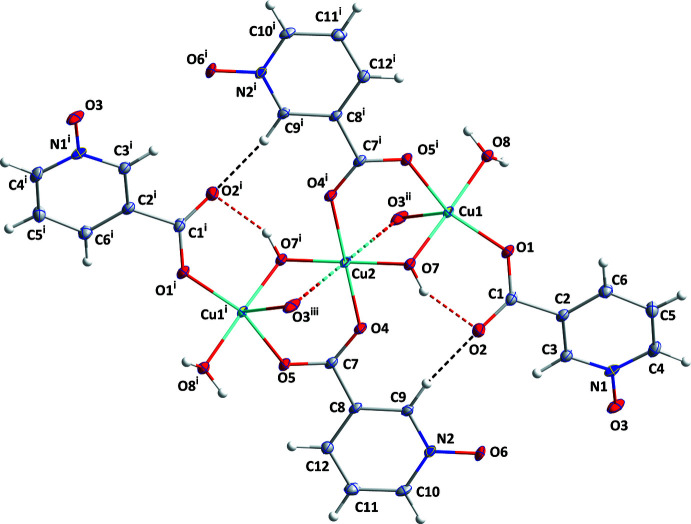
A portion of the title mol­ecule showing the coordination spheres of the independent copper ions with labeling scheme and 50% probability ellipsoids [symmetry codes: (i) −*x* + 1, −*y* + 1, −*z* + 1; (ii) *x* + 1, *y* + 1, *z*; (iii) −*x*, −*y*, −*z* + 1]. O—H⋯O and C—H⋯O hydrogen bonds are depicted, respectively, by red and black dashed lines while the weak Cu2⋯O3^ii^ and Cu2⋯O3^iii^ inter­actions are depicted by aqua/red dashed lines.

The monomer unit plus one *N*-oxide atom from the bridging nicotinato-*N*-oxido ligand on each end copper atom (O3^ii^ and O3^iii^) is shown in Fig. 1 ▸. This moiety is centrosymmetric with Cu2 lying on the crystallographic center of symmetry. The coordination about Cu1 is square pyramidal with the *N*-oxide atom from the bridging nicotinato-*N*-oxido ligand (O3^ii^) in the apical site and the basal sites occupied by the bridging hydroxide (O7—H7) and the water mol­ecule (O8) in *trans* positions, and a carboxyl­ate oxygen atom from the bridging nicotinato-*N*-oxido ligand (O1) and the bridging nicotinato-*N*-oxide ligand (O5^i^). The Cu1—O distances and bond angles are in line with those typically seen for tetra­gonally elongated, square-pyramidal Cu^II^. Cu2 is coordinated by the bridging hydroxide (O7—H7) and a carboxyl­ate oxygen of the nicotinato-*N*-oxide ligand (O4) and their symmetry-related counterparts. Although rigorously planar, the coordination about Cu2 shows a rhombic distortion from square geometry due to the difference in the Cu2—O4 [1.9687 (11) Å] and Cu2—O7 [1.9240 (11) Å] bond lengths and the O4—Cu2—O7 angle of 87.69 (5)°. This geometry is quite comparable to those in the previously reported structures (Table 1 ▸). One feature noted by Kang *et al.* (2020 ▸) but not by Knuutilla (1981 ▸) is a weak contact by the *N*-oxide oxygen atoms coordinated to Cu1 (O3^ii^ and O3^iii^) to Cu2 with the Cu2—O3^ii^ distance of 2.6828 (15) Å being considerably longer than the Cu1—O3^ii^ distance [2.4208 (13) Å] but definitely shorter than the sum of the van der Waals radii (2.92 Å), indicating a short contact. The O7—Cu2—O3^ii^ and O7^i^—Cu2—O3^ii^ angles of 81.66 (5) and 98.34 (5)°, which differ greatly from 90°, suggest the coordination of Cu2 should not be described as an elongated octa­hedron. There are close to 100 structures listed in the CSD (Groom *et al.*, 2016 ▸) with Cu—O distances of 2.69 Å or greater and we cite three examples close to those observed here: 2.693 (4) Å (Laborda *et al.*, 2004 ▸), 2.757 (5) Å (Laza­rou *et al.*, 2018 ▸) and 2.696 (3) Å (Procházková *et al.*, 2017 ▸). In these, the first involves a coordinated water mol­ecule while in the latter two, the distance is to a ligand oxygen atom bridging copper centers and so more comparable to the present work. Where commented on, the long distance is attributed to a Jahn–Teller distortion, but in our case the Cu2—O3^ii^ distance not only is long, but also its direction is tilted away from the Cu2 coordination plane normal by ∼8°. This suggests that O3^ii^ is close to Cu2 for sterical convenience, not due to the formation of a Cu2—O3^ii^ bond. The intra­molecular O7—H7*A*⋯O2 hydrogen bond (Table 2 ▸) belongs to a 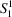
(6) graph set (Bernstein *et al.*, 1995 ▸).

## Supra­molecular features   

**Figure 2:**
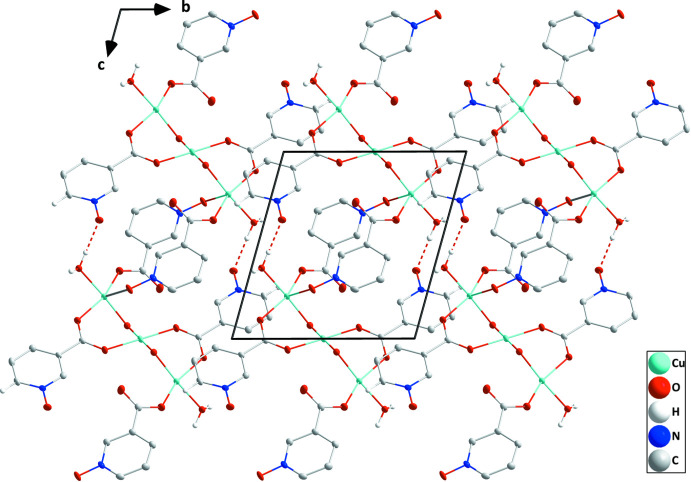
Plan view of one layer seen along the *a*-axis direction with O—H⋯O hydrogen bonds depicted by dashed lines.

**Figure 3:**
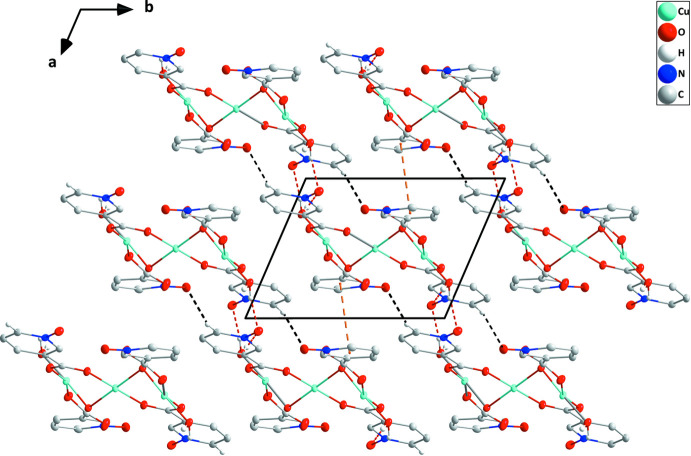
Elevation view of a portion of two layers seen along the *c*-axis direction and showing the π-stacking inter­actions (orange dashed lines) holding them together. O—H⋯O and C—H⋯O hydrogen bonds within layers are depicted by red and black dashed lines, respectively.

The monomer units, [Cu_3_(μ-OH)_2_(H_2_O)_2_(μ-nicNO)_4_], are connected into chains extending along the *c*-axis direction by coordination of *N*-oxide oxygen atom O3 to atom Cu1^i^ of the next unit (Fig. 1 ▸). The chains are linked into layers parallel to (1
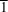
0) by pairwise O8—H8*A*⋯O6^i^ hydrogen bonds [Table 2 ▸ and Fig. 2 ▸; graph-set 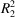
(9)] together with offset π-stacking between inversion-related C2/C3/N1/C4/C5/C6 rings [centroid–centroid = 3.4753 (13) Å, slippage = 0.53 Å] and inversion-related N2/C9/C8/C12/C11/C10 rings [centroid–centroid = 3.6432 (12) Å, slippage = 1.5 Å] (Fig. 3 ▸). The O8—H8*B*⋯O6^ii^ hydrogen bond (Table 2 ▸) is part of a 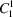
(11) graph set through O4^ii^, Cu2^ii^, Cu1^ii^ and O8^ii^ [symmetry code: (ii) *x* + 1, *y* + 1, *z*) as well as a 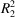
(18) graph set through O5^ii^, Cu2^ii^, Cu1^iv^, O8^iv^ and O6^i^ [symmetry codes: (i) −*x* + 1, −*y* + 1, −*z* + 1; (iv) −*x* + 2, −*y* + 2, −*z*) and a 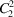
(22) graph set through O4^ii^, Cu2^ii^, O4^v^, O6^v^, O8^vi^, Cu1^vi^, Cu2^vi^, Cu1^vii^ and O8^vii^ [symmetry codes: (v) 2 − *x*, 2 − *y*, −1 − *z*; (vi) 1 + *x*, 1 + *y*, −1 + *z*; (vii) 2 − *x*, 2 − *y*, −1 − *z*).

## Database survey   

A search of the Cambridge Crystallographic Database (CSD, Version 5.41 updated to March 2020; Groom *et al.*, 2016) using the fragments 2-, 3- and 4-carb­oxy­pyridine-*N*-oxide yielded 20 hits, of which 16 were complexes of 4-carb­oxy­pyridine-*N*-oxide, three contained 3-carb­oxy­pyridine-*N*-oxide, including the prior report of the title compound, and one contained 2-carb­oxy­pyridine-*N*-oxide. The last (IJOHAR; Wang *et al.*, 2011 ▸) is also a polymeric Cu^II^ complex in which the organic ligand chelates through one carboxyl­ate oxygen and the *N*-oxide oxygen and bridges to two adjacent metals through the other carboxyl­ate oxygen and the *N*-oxide oxygen. The other two complexes of 3-carb­oxy­pyridine-*N*-oxide are [Dy(H_2_O)(3-carb­oxy­pyridine-*N*-oxide)(squarate)]_*n*_ (OXO­ROK; Liu *et al.*, 2016 ▸) in which the 3-carb­oxy­pyridine-*N*-oxide chelates to one metal through the carboxyl group and bridges to a second through the *N*-oxide oxygen and [Tb_2_(3-carb­oxy­pyridine-*N*-oxide)_4_(H_2_O)_2_(oxalate)]_*n*_ (QUBKEF; Yu *et al.*, 2015 ▸). The complexes of 4-carb­oxy­pyridine-*N*-oxide include a dinuclear Cu^II^ complex containing bidentate bridg­ing and monodentate carboxyl­ate ligands in which the *N*-oxide oxygen is not coordinated (BULWIO; Knuutilla, 1983 ▸) and a polymeric Cu^II^ complex in which all three oxygen atoms of the carboxyl­ate ligand are involved in bridging coord­in­ation modes (YISLAQ; Ghosh *et al.*, 2018 ▸).

## Hirshfeld surface analysis   

**Figure 4:**
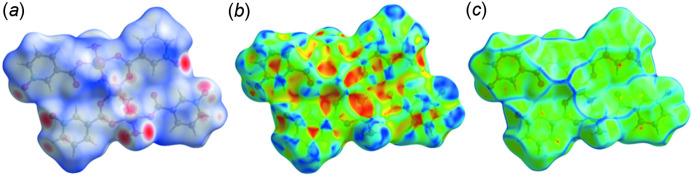
The Hirshfeld surface plotted over (*a*) *d*_norm_, (*b*) shape index and (*c*) curvature.

**Figure 5:**
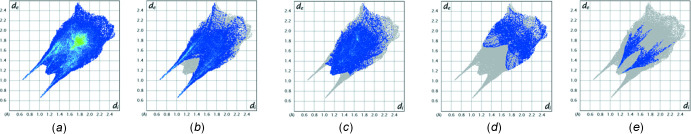
Fingerprint plots showing (*a*) all inter­molecular inter­actions and resolved into (*b*) H⋯O/O⋯H, (*c*) H⋯H, (*d*) H⋯C/C⋯H and (*e*) O⋯Cu/Cu⋯O contacts.

An effective means of probing inter­molecular inter­actions is Hirshfeld surface analysis (McKinnon *et al.*, 2007 ▸; Spackman & Jayatilaka, 2009 ▸), which can be conveniently carried out with *Crystal Explorer 17* (Turner *et al.*, 2017 ▸). A detailed description of the use of *Crystal Explorer 17* and the plots obtained has been published (Tan *et al.*, 2019 ▸) so will not be given here. Fig. 4 ▸ *a* presents the surface mapped over *d*_norm_ over the range −0.7162 to 1.5102 arbitrary units in which the bright-red spots indicate the strong O—H⋯O hydrogen bonds and the lighter red spots the weaker C—H⋯O hydrogen bonds listed in Table 2 ▸. Mapping of the Hirshfeld surface over shape-index is illustrated in Fig. 4 ▸ *b* and provides a picture of possible π-stacking inter­actions. These are indicated by red–orange triangles surrounded by blue triangles, which occur over the pyridine rings, confirming the slipped π-stacking inter­action discussed in Section 3. This is also indicated by the surface mapped over curvature (Fig. 4 ▸ *c*) where the substanti­ally flat regions of the plot again occur over the pyridine rings. Parsing the inter­molecular inter­actions into specific types is accomplished with the fingerprint plots (Fig. 5 ▸). Fig. 5 ▸ *a* shows the full fingerprint plot while Fig. 5 ▸ *b* presents the H⋯O/O⋯H inter­actions which, not surprisingly, constitute the largest of the inter­molecular inter­actions at 35.8% of the total. These are followed by H⋯H (Fig. 5 ▸ *c*, 25.9%), H⋯C/C⋯H (Fig. 5 ▸ *d*, 10.8%) and O⋯Cu (Fig. 5 ▸ *e*, 10.8%) inter­actions. Not shown are the C⋯C (7.9%) and H⋯N/N⋯H (2.5%) contacts, with the former corresponding primarily to the slipped π-stacking inter­actions.

## Synthesis and crystallization   

An aqueous solution of CuCl_2_·2H_2_O (0.034 g, 0.2 mmol in 3.5 mL) was added to an aqueous solution (3.5 mL) containing pyridine-2,3-di­carb­oxy­lic acid (0.04 g, 0.2 mmol) and NaOH (0.2 ml, 1 mol L^−1^), the mixture was stirred at 333 K for 2 h and then cooled to room temperature. After standing for a week, the light-blue precipitate that formed was filtered off and dried. Dark-blue, block-like crystals were obtained by slow evaporation of a solution of the precipitate in 5 mL of distilled water at room temperature. (yield: 30.61% based on Cu). Analysis calculated for: C, 27.00; H, 1.94; N, 4.50%. Found: C, 26.86; H, 2.02; N, 4.46%. IR (cm^−1^ KBr): 445, 489, 547, 612, 674, 688, 768, 798, 948, 1019, 1044, 1130, 1225, 1376, 1408, 1441, 1564, 1594, 1619, 2994, 3043, 3069, 3355.

## Refinement   

Crystal data, data collection and structure refinement details are summarized in Table 3 ▸. H atoms attached to carbon were placed in idealized locations (C—H = 0.95 Å) and were included as riding contributions with *U*_iso_(H) = 1.2*U*_eq_(C). Those attached to oxygen were placed in locations obtained from a difference map and were refined with DFIX O—H = 0.84 (1) Å restraints.

## Supplementary Material

Crystal structure: contains datablock(s) global, I. DOI: 10.1107/S2056989021002000/jq2004sup1.cif

Structure factors: contains datablock(s) I. DOI: 10.1107/S2056989021002000/jq2004Isup2.hkl

CCDC reference: 2063970

Additional supporting information:  crystallographic information; 3D view; checkCIF report

## Figures and Tables

**Table 1:** Comparison of structures (Å,°)

Metric	This work	NICTCU	NICTCU01
Cu1—O1	1.9542 (12)	1.943 (2)	1.953 (3)
Cu1—O7	1.9003 (12)	1.925 (1)	1.893 (4)
Cu1—O8	1.9539 (12)	1.876 (1)	1.947 (4)
Cu1—O5^i^	1.9911 (12)	1.979 (2)	1.987 (3)
Cu1—O3^ii^	2.4208 (13)	2.426 (2)	2.434 (4)
Cu2—O4	1.9687 (11)	1.954 (1)	1.981 (3)
Cu2—O7	1.9240 (11)	1.912 (2)	1.922 (4)
Cu2—O3^ii^	2.6828 (15)		2.699 (3)
			
O1—Cu1—O5^i^	158.98 (5)	158.73 (7)	158.83 (16)
O1—Cu1—O7	97.79 (5)	97.55 (6)	97.87 (14)
O1—Cu1—O8	84.60 (5)	84.84 (6)	84.72 (15)
O7—Cu1—O8	176.16 (5)	176.28 (6)	176.18 (14)
O7—Cu1—O5^i^	92.38 (5)	92.27 (5)	92.13 (14)
O1—Cu1–O3^ii^	103.19 (5)	103.56 (7)	103.40 (15)
O7—Cu1—O3^ii^	89.57 (5)	89.84 (6)	89.24 (15)
O8—Cu1–O3^ii^	92.82 (5)	92.37 (6)	92.89 (15)
O5^i^—Cu1—O3^ii^	95.20 (5)	92.56 (7)	95.31 (14)
O4—Cu2—O4^i^	180.00 (8)		
O7—Cu2—O4^i^	87.69 (5)	87.28 (7)	87.79 (15)
O4—Cu2—O7^i^	92.31 (5)	92.72 (7)	92.21 (15)

Symmetry codes: (i) −*x* + 1, −*y* + 1, −*z* + 1; (ii) *x* + 1, *y* + 1, *z*.

**Table 2:** Hydrogen-bond geometry (Å, °)

*D*—H⋯*A*	*D*—H	H⋯*A*	*D*⋯*A*	*D*—H⋯*A*
O7—H7*A*⋯O2	0.82 (1)	2.04 (1)	2.8057 (17)	156 (2)
O8—H8*A*⋯O6^i^	0.83 (1)	1.84 (1)	2.6684 (17)	173 (3)
O8—H8*B*⋯O6^ii^	0.84 (1)	1.88 (1)	2.6976 (18)	168 (3)
C9—H9⋯O2	0.95	2.28	3.216 (2)	168
C10—H10⋯O3^iii^	0.95	2.23	3.079 (2)	148

Symmetry codes: (i) -x+1, -y+1, -z+1; (ii) x+1, y+1, z; (iii) -x, -y, -z+1.

**Table 3:** Experimental details

Crystal data	
Chemical formula	[Cu_3_(C_6_H_4_NO_3_)_4_(OH)_2_(H_2_O)_2_]
*M*_r_	813.07
Crystal system, space group	Triclinic, *P*\overline{1}
Temperature (K)	150
*a*, *b*, *c* (Å)	7.8669 (17), 9.710 (2), 10.424 (2)
α, β, γ (°)	97.016 (3), 110.701 (3), 109.049 (3)
*V* (Å^3^)	678.2 (2)
*Z*	1
Radiation type	Mo *K*α
μ (mm^−1^)	2.42
Crystal size (mm)	0.31 × 0.25 × 0.22

Data collection	
Diffractometer	Bruker *SMART* *APEX* CCD
Absorption correction	Multi-scan (*SADABS*; Krause *et al.*, 2015 ▸)
*T*_min_, *T*_max_	0.50, 0.62
No. of measured, independent and observed [*I* > 2σ(*I*)] reflections	12893, 3592, 3390
*R*_int_	0.021
(sin θ/λ)_max_ (Å^−1^)	0.685

Refinement	
*R*[*F* ^2^ > 2σ(*F* ^2^)], *wR*(*F* ^2^), *S*	0.023, 0.065, 1.06
No. of reflections	3592
No. of parameters	226
No. of restraints	3
H-atom treatment	H atoms treated by a mixture of independent and constrained refinement
Δρ_max_, Δρ_min_ (e Å^−3^)	0.52, −0.31

Computer programs: *APEX3* and *SAINT* (Bruker, 2016 ▸), *SHELXT* (Sheldrick, 2015*a* ▸), *SHELXL2018/1* (Sheldrick, 2015*b* ▸), *DIAMOND* (Brandenburg & Putz, 2012 ▸) and *SHELXTL* (Sheldrick, 2008 ▸).

## supplementary crystallographic information

### Crystal data

**Table:**

[Cu_3_(C_6_H_4_NO_3_)_4_(OH)_2_(H_2_O)_2_]	*Z* = 1
*M**_r_* = 813.07	*F*(000) = 409
Triclinic, *P*1	*D*_x_ = 1.991 Mg m^−^^3^
*a* = 7.8669 (17) Å	Mo *K*α radiation, λ = 0.71073 Å
*b* = 9.710 (2) Å	Cell parameters from 9998 reflections
*c* = 10.424 (2) Å	θ = 2.3–29.1°
α = 97.016 (3)°	µ = 2.42 mm^−^^1^
β = 110.701 (3)°	*T* = 150 K
γ = 109.049 (3)°	Block, blue
*V* = 678.2 (2) Å^3^	0.31 × 0.25 × 0.22 mm

### Data collection

**Table:**

Bruker SMART APEX CCD diffractometer	3592 independent reflections
Radiation source: fine-focus sealed tube	3390 reflections with *I* > 2σ(*I*)
Graphite monochromator	*R*_int_ = 0.021
Detector resolution: 8.3333 pixels mm^-1^	θ_max_ = 29.2°, θ_min_ = 2.2°
φ and ω scans	*h* = −10→10
Absorption correction: multi-scan (*SADABS*; Krause *et al.*, 2015)	*k* = −13→13
*T*_min_ = 0.50, *T*_max_ = 0.62	*l* = −14→14
12893 measured reflections	

### Refinement

**Table:**

Refinement on *F*^2^	Primary atom site location: dual
Least-squares matrix: full	Secondary atom site location: difference Fourier map
*R*[*F*^2^ > 2σ(*F*^2^)] = 0.023	Hydrogen site location: mixed
*wR*(*F*^2^) = 0.065	H atoms treated by a mixture of independent and constrained refinement
*S* = 1.06	*w* = 1/[σ^2^(*F*_o_^2^) + (0.0347*P*)^2^ + 0.5335*P*] where *P* = (*F*_o_^2^ + 2*F*_c_^2^)/3
3592 reflections	(Δ/σ)_max_ = 0.001
226 parameters	Δρ_max_ = 0.52 e Å^−^^3^
3 restraints	Δρ_min_ = −0.31 e Å^−^^3^

### Special details

**Table:**

Experimental. The diffraction data were obtained from 3 sets of 400 frames, each of width 0.5° in ω, colllected at φ = 0.00, 90.00 and 180.00° and 2 sets of 800 frames, each of width 0.45° in φ, collected at ω = –30.00 and 210.00°. The scan time was 5 sec/frame.
Geometry. All esds (except the esd in the dihedral angle between two l.s. planes) are estimated using the full covariance matrix. The cell esds are taken into account individually in the estimation of esds in distances, angles and torsion angles; correlations between esds in cell parameters are only used when they are defined by crystal symmetry. An approximate (isotropic) treatment of cell esds is used for estimating esds involving l.s. planes.
Refinement. Refinement of F^2^ against ALL reflections. The weighted R-factor wR and goodness of fit S are based on F^2^, conventional R-factors R are based on F, with F set to zero for negative F^2^. The threshold expression of F^2^ > 2sigma(F^2^) is used only for calculating R-factors(gt) etc. and is not relevant to the choice of reflections for refinement. R-factors based on F^2^ are statistically about twice as large as those based on F, and R- factors based on ALL data will be even larger. H-atoms attached to carbon were placed in calculated positions (C—H = 0.95 Å) while those attached to oxygen were placed in locations derived from a difference map and their coordinates adjusted to give O—H = 0.84 %A. The former were included as riding contributions with isotropic displacement parameters 1.2 times those of the attached atoms while the latter were refined subject to the restraint DFIX 0.84 (1).

### Fractional atomic coordinates and isotropic or equivalent isotropic displacement parameters (Å^2^)

**Table:**

	*x*	*y*	*z*	*U*_iso_*/*U*_eq_	
Cu1	0.55161 (3)	0.76197 (2)	0.22674 (2)	0.01203 (6)	
Cu2	0.500000	0.500000	0.000000	0.01188 (7)	
O1	0.44050 (18)	0.71790 (14)	0.36494 (13)	0.0175 (2)	
O2	0.2406 (2)	0.47108 (14)	0.27160 (14)	0.0245 (3)	
O3	0.22121 (19)	0.36322 (14)	0.73304 (14)	0.0189 (2)	
O4	0.38030 (18)	0.32089 (13)	0.05837 (12)	0.0146 (2)	
O5	0.35598 (18)	0.12662 (13)	−0.09985 (12)	0.0154 (2)	
O6	0.09294 (18)	0.09404 (14)	0.36357 (12)	0.0166 (2)	
O7	0.35789 (17)	0.59314 (13)	0.07123 (12)	0.0126 (2)	
H7A	0.308 (3)	0.534 (2)	0.110 (2)	0.029 (6)*	
O8	0.74783 (18)	0.94425 (13)	0.37813 (12)	0.0152 (2)	
H8A	0.788 (4)	0.931 (3)	0.4591 (16)	0.041 (8)*	
H8B	0.848 (3)	0.981 (3)	0.362 (3)	0.035 (7)*	
N1	0.2291 (2)	0.47710 (16)	0.67356 (14)	0.0135 (3)	
N2	0.13829 (19)	0.03830 (15)	0.26216 (14)	0.0119 (2)	
C1	0.3226 (2)	0.59140 (18)	0.36527 (17)	0.0138 (3)	
C2	0.2845 (2)	0.59731 (18)	0.49796 (16)	0.0122 (3)	
C3	0.2577 (2)	0.47220 (18)	0.55191 (17)	0.0136 (3)	
H3	0.259212	0.383122	0.504107	0.016*	
C4	0.2081 (2)	0.59753 (19)	0.73601 (17)	0.0155 (3)	
H4	0.178573	0.595901	0.816802	0.019*	
C5	0.2293 (3)	0.7225 (2)	0.68303 (17)	0.0171 (3)	
H5	0.212768	0.806108	0.726578	0.020*	
C6	0.2751 (2)	0.72616 (19)	0.56552 (17)	0.0152 (3)	
H6	0.299241	0.814314	0.532209	0.018*	
C7	0.3409 (2)	0.18427 (17)	0.00894 (16)	0.0111 (3)	
C8	0.2615 (2)	0.07659 (17)	0.08634 (15)	0.0109 (3)	
C9	0.2181 (2)	0.13269 (17)	0.19519 (16)	0.0117 (3)	
H9	0.244784	0.237223	0.221998	0.014*	
C10	0.0996 (2)	−0.11165 (18)	0.22703 (17)	0.0143 (3)	
H10	0.043095	−0.176028	0.275773	0.017*	
C11	0.1424 (2)	−0.17042 (18)	0.12053 (18)	0.0160 (3)	
H11	0.115782	−0.275146	0.096237	0.019*	
C12	0.2246 (2)	−0.07639 (18)	0.04886 (17)	0.0141 (3)	
H12	0.254937	−0.115727	−0.024337	0.017*	

### Atomic displacement parameters (Å^2^)

**Table:**

	*U*^11^	*U*^22^	*U*^33^	*U*^12^	*U*^13^	*U*^23^
Cu1	0.01623 (11)	0.01106 (10)	0.01109 (10)	0.00360 (8)	0.00955 (8)	0.00412 (7)
Cu2	0.01632 (14)	0.01104 (13)	0.01417 (13)	0.00581 (10)	0.01150 (10)	0.00645 (10)
O1	0.0224 (6)	0.0161 (5)	0.0160 (5)	0.0030 (5)	0.0142 (5)	0.0056 (4)
O2	0.0389 (8)	0.0147 (6)	0.0243 (6)	0.0046 (5)	0.0241 (6)	0.0038 (5)
O3	0.0230 (6)	0.0195 (6)	0.0238 (6)	0.0098 (5)	0.0156 (5)	0.0165 (5)
O4	0.0201 (6)	0.0122 (5)	0.0169 (5)	0.0053 (4)	0.0138 (5)	0.0070 (4)
O5	0.0228 (6)	0.0136 (5)	0.0134 (5)	0.0048 (5)	0.0131 (5)	0.0053 (4)
O6	0.0206 (6)	0.0191 (6)	0.0132 (5)	0.0050 (5)	0.0132 (5)	0.0046 (4)
O7	0.0149 (5)	0.0125 (5)	0.0134 (5)	0.0043 (4)	0.0099 (4)	0.0044 (4)
O8	0.0211 (6)	0.0128 (5)	0.0131 (5)	0.0036 (5)	0.0113 (5)	0.0045 (4)
N1	0.0124 (6)	0.0157 (6)	0.0148 (6)	0.0050 (5)	0.0073 (5)	0.0090 (5)
N2	0.0125 (6)	0.0144 (6)	0.0099 (5)	0.0036 (5)	0.0069 (5)	0.0048 (5)
C1	0.0169 (7)	0.0156 (7)	0.0157 (7)	0.0087 (6)	0.0110 (6)	0.0075 (6)
C2	0.0118 (7)	0.0144 (7)	0.0123 (6)	0.0045 (6)	0.0070 (5)	0.0053 (5)
C3	0.0151 (7)	0.0144 (7)	0.0151 (7)	0.0060 (6)	0.0096 (6)	0.0059 (6)
C4	0.0158 (7)	0.0217 (8)	0.0118 (6)	0.0077 (6)	0.0078 (6)	0.0061 (6)
C5	0.0222 (8)	0.0187 (8)	0.0148 (7)	0.0106 (6)	0.0102 (6)	0.0047 (6)
C6	0.0190 (8)	0.0150 (7)	0.0153 (7)	0.0076 (6)	0.0096 (6)	0.0074 (6)
C7	0.0107 (7)	0.0135 (7)	0.0111 (6)	0.0041 (5)	0.0064 (5)	0.0060 (5)
C8	0.0110 (7)	0.0128 (7)	0.0105 (6)	0.0037 (5)	0.0063 (5)	0.0061 (5)
C9	0.0126 (7)	0.0116 (6)	0.0110 (6)	0.0025 (5)	0.0065 (5)	0.0046 (5)
C10	0.0142 (7)	0.0141 (7)	0.0172 (7)	0.0043 (6)	0.0087 (6)	0.0095 (6)
C11	0.0177 (8)	0.0128 (7)	0.0208 (8)	0.0064 (6)	0.0100 (6)	0.0078 (6)
C12	0.0161 (7)	0.0144 (7)	0.0153 (7)	0.0067 (6)	0.0094 (6)	0.0052 (6)

### Geometric parameters (Å, º)

**Table:**

Cu1—O7	1.9003 (12)	N2—C9	1.3462 (19)
Cu1—O8	1.9539 (12)	N2—C10	1.360 (2)
Cu1—O1	1.9542 (12)	C1—C2	1.513 (2)
Cu1—O5^i^	1.9911 (12)	C2—C3	1.386 (2)
Cu1—O3^ii^	2.4208 (13)	C2—C6	1.396 (2)
Cu2—O7^i^	1.9240 (11)	C3—H3	0.9500
Cu2—O7	1.9240 (11)	C4—C5	1.379 (2)
Cu2—O4	1.9687 (11)	C4—H4	0.9500
Cu2—O4^i^	1.9688 (11)	C5—C6	1.395 (2)
O1—C1	1.276 (2)	C5—H5	0.9500
O2—C1	1.232 (2)	C6—H6	0.9500
O3—N1	1.3270 (17)	C7—C8	1.507 (2)
O4—C7	1.2541 (19)	C8—C12	1.393 (2)
O5—C7	1.2638 (19)	C8—C9	1.394 (2)
O6—N2	1.3381 (17)	C9—H9	0.9500
O7—H7A	0.819 (10)	C10—C11	1.381 (2)
O8—H8A	0.833 (10)	C10—H10	0.9500
O8—H8B	0.836 (10)	C11—C12	1.390 (2)
N1—C4	1.354 (2)	C11—H11	0.9500
N1—C3	1.362 (2)	C12—H12	0.9500
			
O7—Cu1—O8	176.16 (5)	O1—C1—C2	113.52 (14)
O7—Cu1—O1	97.79 (5)	C3—C2—C6	119.94 (14)
O8—Cu1—O1	84.60 (5)	C3—C2—C1	119.12 (14)
O7—Cu1—O5^i^	92.38 (5)	C6—C2—C1	120.94 (14)
O8—Cu1—O5^i^	84.42 (5)	N1—C3—C2	119.92 (15)
O1—Cu1—O5^i^	158.98 (5)	N1—C3—H3	120.0
O7—Cu1—O3^ii^	89.57 (5)	C2—C3—H3	120.0
O8—Cu1—O3^ii^	92.82 (5)	N1—C4—C5	120.41 (15)
O1—Cu1—O3^ii^	103.19 (5)	N1—C4—H4	119.8
O5^i^—Cu1—O3^ii^	95.20 (5)	C5—C4—H4	119.8
O7^i^—Cu2—O7	180.0	C4—C5—C6	119.99 (15)
O7^i^—Cu2—O4	92.31 (5)	C4—C5—H5	120.0
O7—Cu2—O4	87.69 (5)	C6—C5—H5	120.0
O7^i^—Cu2—O4^i^	87.69 (5)	C5—C6—C2	118.49 (15)
O7—Cu2—O4^i^	92.31 (5)	C5—C6—H6	120.8
O4—Cu2—O4^i^	180.00 (8)	C2—C6—H6	120.8
C1—O1—Cu1	128.46 (11)	O4—C7—O5	127.42 (14)
N1—O3—Cu1^ii^	125.82 (9)	O4—C7—C8	116.01 (13)
C7—O4—Cu2	129.98 (10)	O5—C7—C8	116.56 (14)
C7—O5—Cu1^i^	125.97 (10)	C12—C8—C9	119.83 (14)
Cu1—O7—Cu2	106.58 (6)	C12—C8—C7	121.94 (14)
Cu1—O7—H7A	102.7 (18)	C9—C8—C7	118.19 (14)
Cu2—O7—H7A	104.7 (18)	N2—C9—C8	119.81 (14)
Cu1—O8—H8A	115 (2)	N2—C9—H9	120.1
Cu1—O8—H8B	110.4 (19)	C8—C9—H9	120.1
H8A—O8—H8B	106 (3)	N2—C10—C11	120.03 (14)
O3—N1—C4	118.78 (13)	N2—C10—H10	120.0
O3—N1—C3	120.31 (14)	C11—C10—H10	120.0
C4—N1—C3	120.91 (14)	C10—C11—C12	120.01 (15)
O6—N2—C9	118.69 (13)	C10—C11—H11	120.0
O6—N2—C10	119.75 (13)	C12—C11—H11	120.0
C9—N2—C10	121.54 (14)	C11—C12—C8	118.77 (15)
O2—C1—O1	127.26 (15)	C11—C12—H12	120.6
O2—C1—C2	119.22 (14)	C8—C12—H12	120.6
			
Cu1^ii^—O3—N1—C4	−133.95 (13)	Cu2—O4—C7—O5	−5.8 (3)
Cu1^ii^—O3—N1—C3	46.08 (19)	Cu2—O4—C7—C8	175.93 (10)
Cu1—O1—C1—O2	−6.2 (3)	Cu1^i^—O5—C7—O4	0.0 (2)
Cu1—O1—C1—C2	174.06 (10)	Cu1^i^—O5—C7—C8	178.24 (10)
O2—C1—C2—C3	35.0 (2)	O4—C7—C8—C12	−175.48 (15)
O1—C1—C2—C3	−145.22 (15)	O5—C7—C8—C12	6.0 (2)
O2—C1—C2—C6	−144.61 (17)	O4—C7—C8—C9	6.8 (2)
O1—C1—C2—C6	35.1 (2)	O5—C7—C8—C9	−171.71 (14)
O3—N1—C3—C2	−173.87 (14)	O6—N2—C9—C8	−178.18 (13)
C4—N1—C3—C2	6.2 (2)	C10—N2—C9—C8	0.5 (2)
C6—C2—C3—N1	−2.3 (2)	C12—C8—C9—N2	−0.9 (2)
C1—C2—C3—N1	178.07 (14)	C7—C8—C9—N2	176.91 (13)
O3—N1—C4—C5	175.41 (15)	O6—N2—C10—C11	178.72 (14)
C3—N1—C4—C5	−4.6 (2)	C9—N2—C10—C11	0.1 (2)
N1—C4—C5—C6	−0.8 (3)	N2—C10—C11—C12	−0.2 (2)
C4—C5—C6—C2	4.5 (3)	C10—C11—C12—C8	−0.2 (2)
C3—C2—C6—C5	−2.9 (2)	C9—C8—C12—C11	0.8 (2)
C1—C2—C6—C5	176.69 (15)	C7—C8—C12—C11	−176.96 (14)

Symmetry codes: (i) −*x*+1, −*y*+1, −*z*; (ii) −*x*+1, −*y*+1, −*z*+1.

### Hydrogen-bond geometry (Å, º)

**Table:**

*D*—H···*A*	*D*—H	H···*A*	*D*···*A*	*D*—H···*A*
O7—H7*A*···O2	0.82 (1)	2.04 (1)	2.8057 (17)	156 (2)
O8—H8*A*···O6^ii^	0.83 (1)	1.84 (1)	2.6684 (17)	173 (3)
O8—H8*B*···O6^iii^	0.84 (1)	1.88 (1)	2.6976 (18)	168 (3)
C9—H9···O2	0.95	2.28	3.216 (2)	168
C10—H10···O3^iv^	0.95	2.23	3.079 (2)	148

Symmetry codes: (ii) −*x*+1, −*y*+1, −*z*+1; (iii) *x*+1, *y*+1, *z*; (iv) −*x*, −*y*, −*z*+1.
